# Release-Recapture Studies Confirm Dispersal of *Glossina palpalis gambiensis* between River Basins in Mali

**DOI:** 10.1371/journal.pntd.0002022

**Published:** 2013-04-25

**Authors:** Marc J. B. Vreysen, Thomas Balenghien, Khalfan M. Saleh, Sadou Maiga, Zowinde Koudougou, Giuliano Cecchi, Jérémy Bouyer

**Affiliations:** 1 Insect Pest Control Laboratory, Joint Food and Agriculture Organization/International Atomic Energy Agency (FAO/IAEA) Programme of Nuclear Techniques in Food and Agriculture, Vienna, Austria; 2 Centre de coopération internationale en recherche agronomique pour le développement (CIRAD), Unité Mixte de Recherche Contrôle des Maladies Animales Exotiques et Emergentes, Montpellier, France; 3 Institut National de Recherche Agricole, Unité Mixte de Recherche 1309 Contrôle des Maladies Animales Exotiques et Emergentes, Montpellier, France; 4 Ministry of Agriculture, Natural Resources and Environment, Zanzibar, Tanzania; 5 Direction Nationale de l'Appui au Monde Rural, Unité Centrale de Lutte contre les Mouches Tsé-Tsé et les Trypanosomoses Animales, Bamako, Mali; 6 Pan-African Tsetse and Trypanosomosis Eradication Campaign (PATTEC), Projet de Création de Zones Libérées Durablement de Tsé-tsé et de Trypanosomoses (PCZLD), Bobo-Dioulasso, Burkina Faso; 7 Food and Agriculture Organization of the United Nations (FAO), Animal Production and Health Division, Viale delle Terme di Caracalla, Rome, Italy; 8 Institut Sénégalais de Recherches Agricoles, Laboratoire National d'Elevage et de Recherches Vétérinaires, Dakar–Hann, Sénégal

The cotton belt in Mali/Burkina Faso is among the eco-zones with the highest potential for agriculture and livestock development in West Africa. In this zone, most humans live in rural settings and the development of more sustainable and profitable livestock and mixed-farming systems is mainly constrained by African animal trypanosomosis (AAT), transmitted by the riverine tsetse flies *Glossina palpalis gambiensis* Vanderplank and *Glossina tachinoides* Westwood [1].

The Pan African Tsetse and Trypanosomosis Eradication Campaign (PATTEC) recognised that, for tsetse eradication to be sustainable, it requires an area-wide approach where the control effort is directed against an entire pest population within a circumscribed area [2]. Examples of successful and sustainable area-wide integrated pest management (AW-IPM) campaigns against tsetse include the eradication of (i) *Glossina pallidipes* Austen from Zulu Land in South Africa [3], (ii) *Glossina austeni* Newstead from Unguja Island, Zanzibar [2], and (iii) *Glossina morsitans centralis* Machado from the Okavango delta in Botswana [4]. All these areas are to date still tsetse-free. Whereas isolation is relatively easy to ascertain for islands populations, establishing the limits of target populations on mainland Africa is more challenging and modern tools of population genetics and remote sensing can greatly assist in that respect [5], [6].

As riverine tsetse populations are mainly confined to suitable vegetation along the hydrological network in the subhumid savannah, it was suggested that the “river basin” could be used as the unit of operation in AW-IPM [7]. This assumed that each primary river basin (and possibly also secondary and tertiary) contains tsetse populations that are geographically isolated from those belonging to adjacent basins. However, whereas dispersal of *G. p. gambiensis* is mainly linear along the hydrological network during the dry season, flies can also disperse perpendicular to the river systems, especially during the rainy season, although accurate field data are scarce [8].

To clarify the role of river basins in structuring tsetse populations, genetic studies were initiated in various areas in West Africa. These studies indicated considerable gene flow between riverine tsetse populations belonging to different river basins, and hence, these populations cannot be considered isolated [9]–[11]. However, genetics can only give indirect indications that could be confirmed by directly measuring the potential of the flies to cross the watersheds between adjacent river basins. Here, we present the results of a release–recapture study conducted to assist the planning of the PATTEC national project in Mali. Sterile *G. p. gambiensis* were released in tributaries of two river basins (Senegal and Bani), in close proximity to the adjacent basin (Niger). Attempts were made to recapture the released flies in the savannah between the river basins and, in one site, well within the adjacent river basin.

**Figure 1:**
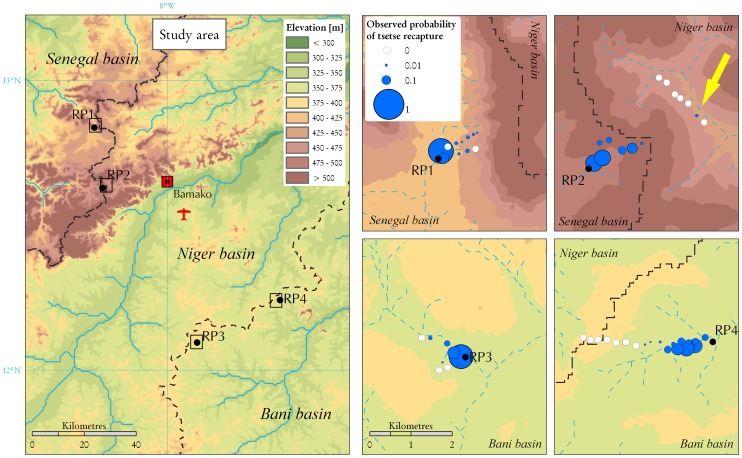
Location of the release points (RPs) and of the trapping sites. The elevation, the boundaries of the three river basins (Senegal, Niger, and Bani), and the hydrological network were derived from the HydroSHEDs dataset.

The flies were sourced from the *G. p. gambiensis* colony maintained at the *Centre International de Recherche-Développement Sur l'Elevage en Zone Subhumide* (CIRDES), Bobo Dioulasso, Burkina Faso since 1972. Flies were sterilised with a dose of 110 Gy in a ^137^Ce source. Both sterile male and female flies were marked with a dot of acrylic paint on the thorax, with a different colour for each week. The flies were transported in carton release containers (dimensions 115×90×50 mm) at a density of 100 flies per box with chartered light aircraft from Bobo Dioulasso to Bamako, Mali, arriving at destination between 7:30 and 10:30 a.m. Upon arrival at the airport, the flies were immediately transported by car to the different release points (RPs). RP 1 and 2 were located on tributaries of the river Senegal and RP 3 and 4 on tributaries of the river Bani (Figure 1). The RPs in the Bani basin were located 83 and 62 km from Bamako airport, and it took 2 h to reach each of them. The RPs in the Senegal basin were located 60 and 65 km from the airport, and it took, respectively, 2.5 h and 1.3 h to reach them.

Fifty-six unbaited biconical traps [12] were deployed between 110 and 3,075 m from the RPs twice a week and collected after 48 h of trapping, for a period of 4 wk. Twelve, 15, 10, and 19 traps were deployed around RP 1, 2, 3, and 4, respectively. A total of 56,000 sterile flies were released at 7-d intervals from 20 July to 10 August 2004—that is, during the rainy season. At each date, 14,000 sterile flies were released, comprising 2,000 males and 1,500 females per site.

As the daily catches were not collected by the field teams, no data are available to estimate the mortality of the released flies. We thus compared observations to the cumulative recapture rates at different distances from the release sites obtained by simulating a two-dimensional random walk with a daily displacement λ between 100 and 1,000 m (increment of 10 m) and a constant mortality rate μ of 0.1 (0.07–0.14) for the entire observation period (28 d). The confidence intervals below corresponded to the values giving the same maximal correlation coefficient using this mortality range. Similar mortality rates were observed for this strain of *G. p. gambiensis* in Burkina Faso [13] and Senegal (Bouyer, unpublished data). Then, we used the best estimation of λ (estimated as the one maximising the correlation between observed and predicted probabilities) and a mortality rate closer to that of natural tsetse populations (0.02, C.I. 0.01–0.03) [14] to assess the mean dispersal distance by generation (mean of the absolute displacement of all individuals in the population), which can be compared to inferences made by population genetics methods (see [13] for details).

During the entire monitoring period, a total of 589 sterile male flies (1.8% recapture rate) and 327 sterile female flies (1.4% recapture rate) were trapped. In addition, a total of 18 wild males and 16 wild females were caught (apparent density of 0.020 male flies/trap/d and 0.018 female flies/trap/d). Mean survival upon arrival at the RPs was 70% and 85% for the sterile male and female flies, respectively. Percentage of nonfliers were 0% and 7%, respectively. In one of the two sites where traps were placed also in the adjacent Niger basin (RP2), one marked female fly was recaptured on the other side of the watershed (Figure 1).

The estimations of λ were very similar between batches and sites, but surprisingly, it was higher in females (mean 780 m, C.I. 760–910) than males (450 m, C.I. 440–670 m), corresponding to diffusion coefficients D of 0.304 km^2^/d and 0.101 km^2^/d (D = λ^2^/2). With an average mortality rate of 0.02 (natural populations) and using the mean estimation of λ, the calculated average dispersal distances were 1,268 m for female and 501 m for male flies.

**Figure 2:**
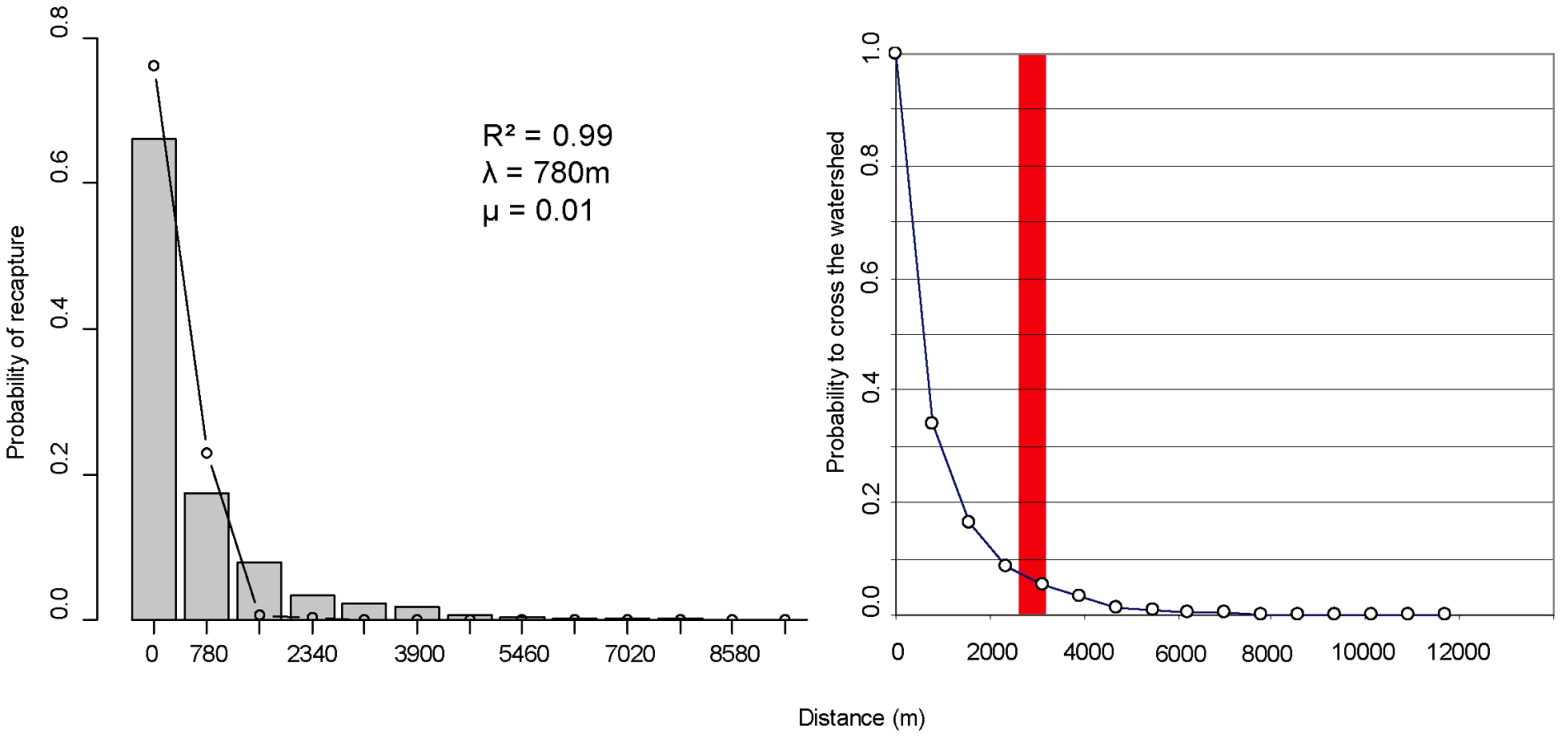
Diffusion probabilities of *Glossina palpalis gambiensis* released in Mali. (Left) Observed cumulated probabilities of presence of all series of females (circles) and predicted probability distributions from an isotropic 2D random walk at various distances from the release points, and corresponding mortality (μ), mean square displacement (λ), and correlation coefficient between observed and predicted (R^2^) (bars). (Right) Probability of a fly to cross the watershed divide in function of its distance to the release point (the red area corresponds to the average distance between RP 3 and the respective traps placed in the Niger Basin).

Since the average distance between the release point RP 2 and the traps deployed in the adjacent Niger Basin was ∼3 km, the probability that one fly might reach one of the traps was 0.086, for λ = 780 m and μ = 0.1 (Figure 2). As 5,179 flies were released in this site of which 41 flies were recaptured (0.8%), and with 7 of the 15 traps deployed on the other side of the watershed, it was expected to trap 1.66 flies at this side, which is close to the actual trap rate of 1.

The data presented in this paper indicate that 110 Gy-treated flies (which can be considered of inferior biological quality as compared to their native counterparts) were capable of crossing the watershed between adjacent river basins in Mali. Although only one fly was recaptured in the adjacent river basin, it is proof of principle that *G. p. gambiensis* can disperse between river basins in Mali. The dispersal data are comparable to the measured diffusion coefficients in savannah areas, and flies were recaptured in traps deployed at 2 km distance from the river forest in Burkina Faso [8] and at ≈2.9 km from the release point in the present study (a female). The mean dispersal distances observed here are much higher than those estimated between river basins in Burkina Faso using population genetics (19–26 m) [10] but in line with those estimated from release–recapture studies along the main Mouhoun river (153–1,053 m) [13]. This is probably due to the fact that two different aspects were measured in the two studies: the dispersal of flies artificially released in unfavourable sites in the current study (as confirmed by the very low density of wild flies), which will induce the flies to disperse quicker to suitable sites for resting or larvipositioning, and the natural dispersal of flies in the previous studies.

These data corroborate results from population genetics studies indicating that in West Africa *G. p. gambiensis* populations from different river basins cannot be considered isolated from one another. Barriers to prevent reinvasion would have to be established between eradication blocks should governments involved in the PATTEC initiative plan a sequential eradication strategy using the rolling carpet approach [15]. It was previously reported that deltamethrin-treated biconical traps deployed at 100 m intervals in riparian forest along a 7 km river section prevented migration of *G. p. gambiensis* and *G. tachinoides* [16]. More recently, insecticide-impregnated cloth targets deployed at ∼250 m intervals forming a barrier with a width between 2 and 25 km successfully prevented reinvasion of *G. m. centralis* in the Okavango delta in Botswana [4]. These barriers can be reinforced using insecticide-treated cattle [17]. However, most of these barriers have been shown to have a very low temporal efficacy as they require proper maintenance, and in most cases, they have proven not to be sustainable [18]. Barriers that are temporarily deployed to prevent reinvasion between intervention blocks to protect achievements made in each phase can be very valuable when used in eradication programmes that proceed in phases or blocks and that have a progressing eradication front. However, if an eradication strategy is not selected or not feasible, then a long-term suppression approach, where farmers themselves can apply control tactics such as localised insecticide treatment of cattle [19], is probably a good alternative to alleviate the burden of animal trypanosomosis. Further population genetics studies are being conducted across the entire *G. p. gambiensis* belt from Ghana to Senegal with a view to determining the most appropriate locations for establishing barriers to prevent reinvasion, taking into account the suitability and fragmentation of vegetation between the river basins.
